# Interplay between Antibiotic Efficacy and Drug-Induced Lysis Underlies Enhanced Biofilm Formation at Subinhibitory Drug Concentrations

**DOI:** 10.1128/AAC.01603-17

**Published:** 2017-12-21

**Authors:** Wen Yu, Kelsey M. Hallinen, Kevin B. Wood

**Affiliations:** a Department of Physics, University of Michigan, Ann Arbor, Michigan, USA; b Department of Biophysics, University of Michigan, Ann Arbor, Michigan, USA

**Keywords:** Enterococcus, biofilms, mathematical modeling, subinhibitory antibiotics

## Abstract

Subinhibitory concentrations of antibiotics have been shown to enhance biofilm formation in multiple bacterial species. While antibiotic exposure has been associated with modulated expression of many biofilm-related genes, the mechanisms of drug-induced biofilm formation remain a focus of ongoing research efforts and may vary significantly across species. In this work, we investigate antibiotic-induced biofilm formation in Enterococcus faecalis, a leading cause of nosocomial infections. We show that biofilm formation is enhanced by subinhibitory concentrations of cell wall synthesis inhibitors but not by inhibitors of protein, DNA, folic acid, or RNA synthesis. Furthermore, enhanced biofilm is associated with increased cell lysis, increases in extracellular DNA (eDNA) levels, and increases in the density of living cells in the biofilm. In addition, we observe similar enhancement of biofilm formation when cells are treated with nonantibiotic surfactants that induce cell lysis. These findings suggest that antibiotic-induced biofilm formation is governed by a trade-off between drug toxicity and the beneficial effects of cell lysis. To understand this trade-off, we developed a simple mathematical model that predicts changes in antibiotic-induced biofilm formation due to external perturbations, and we verified these predictions experimentally. Specifically, we demonstrate that perturbations that reduce eDNA (DNase treatment) or decrease the number of living cells in the planktonic phase (a second antibiotic) decrease biofilm induction, while chemical inhibitors of cell lysis increase relative biofilm induction and shift the peak to higher antibiotic concentrations. Overall, our results offer experimental evidence linking cell wall synthesis inhibitors, cell lysis, increased eDNA levels, and biofilm formation in E. faecalis while also providing a predictive quantitative model that sheds light on the interplay between cell lysis and antibiotic efficacy in developing biofilms.

## INTRODUCTION

Biofilms are dense, surface-associated, microbial communities that play important roles in infectious diseases and a range of device-related clinical infections (1, 2). Biofilms exhibit a fascinating range of community behavior (3), including long-range metabolic codependence (4) and electrical signaling (5–7), phenotypic phase variation (8) and spatial heterogeneity (9), strong ecological competition (10), and multiple types of cooperative behavior, including collective resistance to antimicrobial therapy (11–13). The biofilm response to antibiotics has been a topic of particular interest, with biofilms across species showing dramatically increased resistance to antibiotics relative to planktonic cells. However, a number of studies have shown, somewhat counterintuitively, that exposure to sublethal doses of antibiotics may enhance biofilm formation in a wide range of species (14–16). While antibiotic-mediated biofilm induction has been associated with modulated expression of biofilm-related genes, particularly those affiliated with bacterial and cell surface adhesion, cell motility, or metabolic stress, the mechanisms vary across species and drug classes and remain a focus of ongoing research efforts (14, 16).

In this work, we investigate the effects of sublethal antibiotic concentrations on biofilm formation by Enterococcus faecalis, Gram-positive bacteria commonly underlying nosocomial infections, including bacteremia, native and prosthetic valve endocarditis, and multiple device infections (1, 17). While our understanding of the molecular basis of both biofilm development and drug resistance in E. faecalis continues to mature rapidly (17, 18), surprisingly little attention has been paid to the impact of subinhibitory antibiotic treatments on E. faecalis communities. However, a recent series of intriguing studies has shown that E. faecalis biofilm formation (without antibiotic) hinges on an interplay between fratricide-associated cell lysis and the release of extracellular DNA (eDNA) (19–23). More generally, eDNA is widely recognized as a critical component of biofilm structure in many species (24–26). Additionally, a recent study in Staphylococcus aureus showed that β-lactams administered at subinhibitory concentrations promoted biofilm formation and induced eDNA release in an autolysin-dependent manner (27). Taken together, these results suggest that, for some drugs, biofilm induction hinges on a balance between the inhibitory effects of antibiotics, which reduce biofilm formation at sufficiently high concentrations, and the potential of antibiotic-induced cell lysis to promote biofilm formation, presumably through release of eDNA. Here we investigate this balance in E. faecalis biofilms exposed to multiple classes of antibiotics. We find that subinhibitory concentrations of cell wall synthesis inhibitors, but not other drug classes, promote biofilm formation associated with increased cell lysis and increased eDNA and extracellular RNA (eRNA) levels. Using a simple mathematical model, we quantify the trade-offs between drug efficacy and “beneficial” cell lysis and we use the model to predict the effects of environmental perturbations, including the addition of DNase or chemical inhibitors of lysis, on the location and magnitude of optimal biofilm production. Our results suggest that inhibitors of cell wall synthesis promote biofilm formation via increased cell lysis, and they offer a quantitative predictive framework for understanding the trade-offs between drug toxicity and lysis-induced biofilm induction.

## RESULTS

### Cell wall synthesis inhibitors, but not other classes of antibiotics, promote biofilm formation at low concentrations.

**FIG 1:**
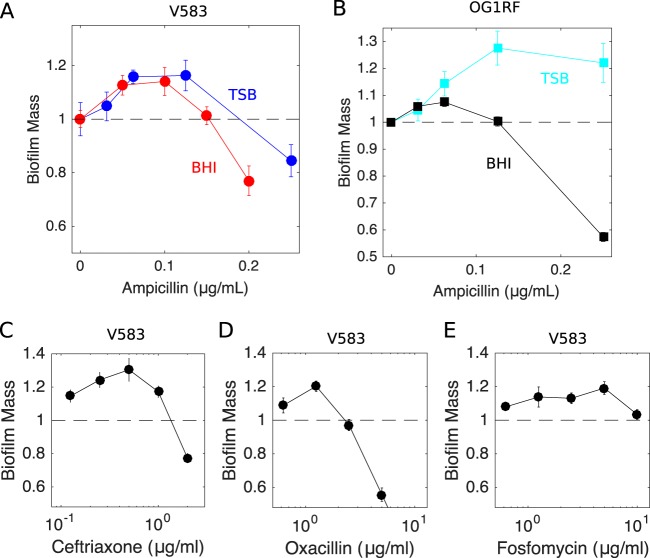
Inhibitors of cell wall synthesis enhance biofilm formation at low concentrations. (A) Biofilm mass (normalized to 1, the value in the absence of drug) as a function of ampicillin concentration for E. faecalis strain V583 in TSB (blue) and BHI medium (red). (B) Similar curves as in panel A, for E. faecalis strain OG1RF in TSB (light blue) and BHI medium (black). (C to E) Similar curves for V583 in BHI medium exposed to three additional cell wall synthesis inhibitors, i.e., ceftriaxone (C), oxacillin (D), and fosftomycin (E). In all panels, biofilm mass was measured with the crystal violet assay (see Materials and Methods). Error bars indicate standard errors of the mean from 6 to 12 replicates.

To investigate antibiotic-induced biofilm formation, we exposed cultures of E. faecalis V583, a fully sequenced clinical isolate, to ampicillin during the first 24 h of biofilm development. Using a bulk crystal violet staining assay (see Materials and Methods), we observed a statistically significant enhancement of biofilm formation after 24 h in the presence of low concentrations of ampicillin (Fig. 1A). Ampicillin at these concentrations has almost no effect on the growth of planktonic cultures, leading to only a slight decrease in the steady-state cell density (see Fig. S1A in the supplemental material). Similar enhancement of biofilm formation was observed for cells grown in different types of medium (brain heart infusion [BHI] medium or tryptic soy broth [TSB]) as well as for strain OG1RF, a common laboratory strain (Fig. 1B), with the magnitude of the enhancement ranging from ∼10% to ∼30%.

**FIG 2:**
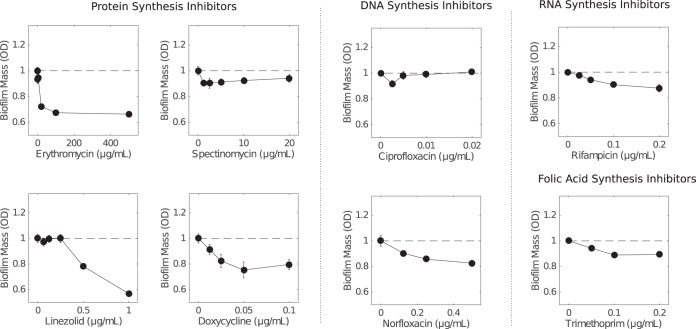
Antibiotics that do not target the cell wall do not enhance biofilm formation at low concentrations. Biofilm mass (normalized to 1, the value in the absence of drug) is shown as a function of antibiotic concentration for E. faecalis strain V583 in BHI medium with exposure to protein synthesis inhibitors (erythromycin, spectinomycin, linezolid, and doxycycline), DNA synthesis inhibitors (ciprofloxacin and norfloxacin), a RNA synthesis inhibitor (rifampin), and a folic acid synthesis inhibitor (trimethoprim). In all panels, biofilm mass was measured with the crystal violet assay (see Materials and Methods). Error bars indicate standard errors of the mean from 6 to 12 replicates.

To determine whether the biofilm enhancement was specific to ampicillin, we performed similar experiments with antibiotics from multiple drug classes. Interestingly, we observed similar increases in biofilm mass with other drugs inhibiting cell wall synthesis, including ceftriaxone, oxacillin, and fosfomycin (Fig. 1C to E), whose mechanism of action is tightly linked to cell lysis. In contrast, drugs targeting protein synthesis, DNA synthesis, RNA synthesis, and folic acid synthesis did not appear to promote biofilm formation over the range of subinhibitory concentrations tested (Fig. 2).

### Biofilm enhancement occurs at subinhibitory concentrations but is associated with increased cell lysis and extracellular nucleic acid levels.

**FIG 3:**
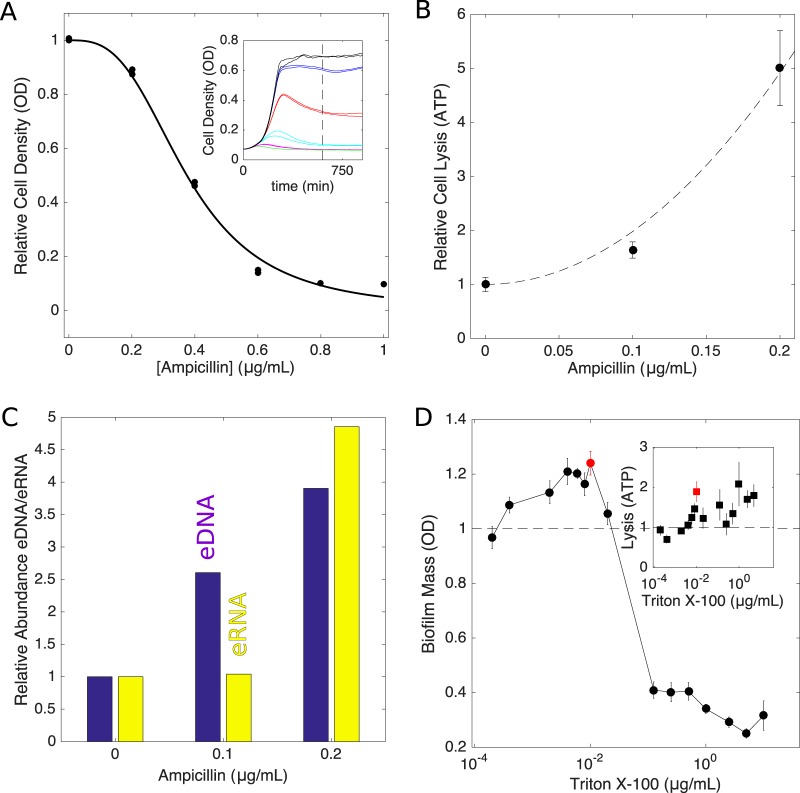
Enhanced biofilm formation occurs at subinhibitory concentrations and is associated with increased cell lysis and increased extracellular nucleic acid. (A) Relative cell density (OD) approximately 10 h after addition of ampicillin. Solid curve, fit to [1 − (*A*/*K*_50_)^*h*^]^−1^, with *A* being the ampicillin concentration, *K*_50_ being 0.38 ± 0.01 μg/ml, the half-maximal inhibitory concentration of the drug, and *h* being 3, a coefficient that describes the steepness of the dose-response curve. Inset, time series of optical density following drug exposure at time zero for ampicillin concentrations of 0 μg/ml (black), 0.2 μg/ml (blue), 0.4 μg/ml (red), 0.6 μg/ml (green), 0.8 μg/ml (magenta), and 1.0 μg/ml (cyan). (B) Cell lysis (relative to untreated cells) as a function of ampicillin concentration, as measured with the ATP luminescence assay (see Materials and Methods). Error bars indicate standard errors of the mean from 8 replicates. Dashed line, fit to 1 + *a*^2^/*r*_00_, with *a* being the ampicillin concentration [measured in units of the drug's half-maximal inhibitory concentration (*K*_50_)] and *r*_00_ being 0.010 ± 0.001. (C) Abundance of eDNA (blue) or eRNA (yellow) as a function of the ampicillin concentration. Abundance was normalized relative to the eDNA (or eRNA) level measured in the absence of drug (also see Fig. S1 in the supplemental material). (D) Triton X-100 (known inducer of cell lysis) enhancement of biofilm formation at low concentrations. Biofilm mass was measured with the crystal violet assay (see Materials and Methods), and error bars indicate standard errors of the mean from 8 replicates. Inset, cell lysis (relative to untreated cells) as a function of Triton X-100 concentration. Red points correspond to peak biofilm formation.

For ampicillin, peak biofilm formation occurs with concentrations of approximately 0.1 μg/ml, which has little effect on the growth of planktonic cell cultures (Fig. S1). To determine the effects of ampicillin on planktonic cultures over a wider drug range, we measured optical density (OD)-time series of V583 cultures exposed to ampicillin concentrations of up to 1 μg/ml (Fig. 3A). Ampicillin has little effect (<15%) on the steady-state density of cells up to concentrations of approximately 0.2 μg/ml, and the dose-response curve is well approximated by a Hill-like function (commonly used in pharmacology [28]) with a half-maximal inhibitory concentration of *K*_50_ = 0.38 ± 0.01 μg/ml. Therefore, increased biofilm formation occurs at concentrations considerably lower than the half-maximal inhibitory concentration measured for planktonic cultures.

While these drug concentrations do not appreciably affect planktonic cell growth, it is possible that they still produce a measurable increase in cell lysis. To investigate this issue, we measured cell lysis in 24-h biofilms (Fig. 3B) and planktonic cultures (Fig. S1) using an ATP-based luminescence assay (29). Indeed, we observed increased cell lysis even with low doses of ampicillin (≤0.2 μg/ml), with lysis increasing nearly 5-fold in biofilms and more than 1,000-fold in planktonic cultures for the highest doses.

Because eDNA has been implicated in E. faecalis biofilm formation, we next asked whether subinhibitory doses of ampicillin lead to increased quantities of extracellular nucleic acids in biofilms. To answer this question, we grew 24-hour biofilms in 5-ml cultures with various concentrations of ampicillin, harvested the biofilms, removed cells by centrifugation, and then extracted nucleic acid from the remaining supernatant. We then quantified DNA or RNA, following treatment with RNase or DNase, respectively, using quantitative imaging after agarose gel electrophoresis (Fig. 3C). Both eDNA and eRNA levels increased with ampicillin treatment, with eDNA (but not eRNA) levels increasing even at the lowest dose (ampicillin at 0.1 μg/ml).

### Nonantibiotic induction of cell lysis promotes biofilm formation.

Because cell lysis is observed at subinhibitory doses of ampicillin and because lysis has been implicated in E. faecalis biofilm formation (19–23), we next asked whether nonantibiotic inducers of cell lysis might also increase biofilm mass at low concentrations. To test this hypothesis, we grew biofilms in the presence of Triton X-100, a surfactant and known inducer of cell lysis (30). Interestingly, we observed enhancement of biofilm formation similar in magnitude (∼20%) to that observed with cell wall inhibitors with Triton X-100 concentrations that yielded similar (∼2-fold) increases in cell lysis (Fig. 3D).

### Antibiotic-induced biofilm formation corresponds to increases in the density of living cells and mean cell area.

**FIG 4:**
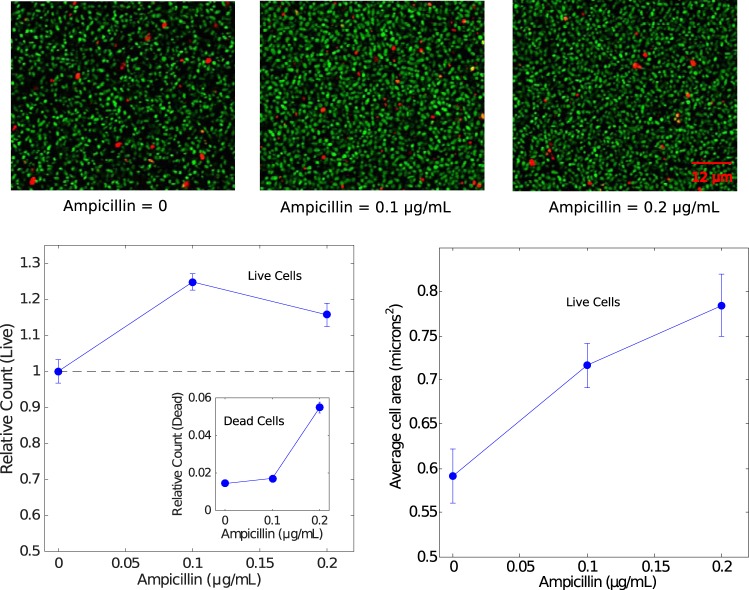
Enhanced biofilm formation corresponds to an increase in the density of living cells. (Top) Example sections from laser scanning confocal images of biofilms exposed to ampicillin at different concentrations (left, 0 μg/ml; middle, 0.1 μg/ml; right, 0.2 μg/ml) and posttreated with live (green) and dead (red) stains. (Lower left) Relative counts of live cells and dead cells (inset) as a function of ampicillin concentration. Counts were normalized relative to the average number of live cells per slice (160 μm by 160 μm) in the absence of drug, which was set to 1; each slice contained on the order of 10^3^ to 10^4^ live cells. (Lower right) Mean area of living cells. Error bars indicate standard errors of the mean taken over a total of 48 two-dimensional slices per condition (three z-slices, taken at identical heights, for each biofilm and 16 independent biofilms per condition). The analysis involves on the order of 10^5^ total live cells per condition.

While our results indicate that biofilm mass is increased with low doses of ampicillin, it is not clear whether this enhancement is due to an increase in the number of living cells or merely an increase in bulk biofilm mass, which may include both viable and nonliving components. To answer this question, we grew 16 replicate biofilms at three different antibiotic concentrations, treated them with live-dead cell stains, and quantified the number of live and dead cells in two-dimensional sections (i.e., the spatial cell density), at single-cell resolution, using laser scanning confocal microscopy (see Materials and Methods). We observed an increase of approximately 25% in the number of living cells per slice, an increase similar in magnitude to the effects observed in bulk experiments (Fig. 4). Interestingly, we also observed a slight increase in the mean size of the living cells as the drug concentration was increased. These results indicate that sections of biofilms formed in the presence of subinhibitory concentrations contain more living cells and more total living cell area, not merely an increase in nonliving mass, compared to those formed in the absence of drug.

### A simple mathematical model describes biofilm induction as a balance between beneficial cell lysis and costly drug efficacy.

To quantify the trade-offs between antibiotic efficacy and beneficial cell lysis, we developed a simple mathematical model describing the mass of living cells (*N*) and the mass of lysed cells and dead cell material (*D*), including eDNA, in a biofilm. Specifically, we have

(1) $$\begin{array}{c} \frac{\partial N}{\partial t}=g\;(1-\frac{N}{K})N-rN+cLD\\ \frac{\partial D}{\partial t}=rN-\gamma D \end{array}$$

In the first equation, the first term describes logistic growth (with per capita growth *g* and carrying capacity *K* >0), the second describes cell death (lysis) with a rate *r* ≥0, and the last describes the increase in biofilm mass due to surface attachment/adhesion of living cells in the planktonic phase (*L*), a process that is coupled to the dead cell mass *D* and is controlled by a parameter *c* >0. Models without this coupling do not exhibit a drug-induced maximum in biofilm mass (see the supplemental material). While the molecular mechanism of coupling is not specified in the model, this term could describe eDNA-mediated attachment and adhesion of planktonic cells, which is assumed here to occur at a rate proportional to both the living cells in solution (*L*) and the lysed cell material in the biofilm (*D*). In the second equation, the first term accounts for cell lysis and the second term describes a decay of lysed cell material (e.g., eDNA) due to, for example, detachment from the biofilm. The model implicitly assumes that the effect of antibiotic on cells in the planktonic phase occurs on a short time scale, allowing *L* to reach a steady state on the time scale of biofilm formation. This assumption is consistent with experimental measurements; planktonic populations reach a steady-state size after approximately 10 h (Fig. 3), while biofilms are formed over a longer 24-h period. The model includes two parameters, *r* = *r*(*a*) and *L* = *L*(*a*), that depend on the drug concentration, *a*.

**FIG 5:**
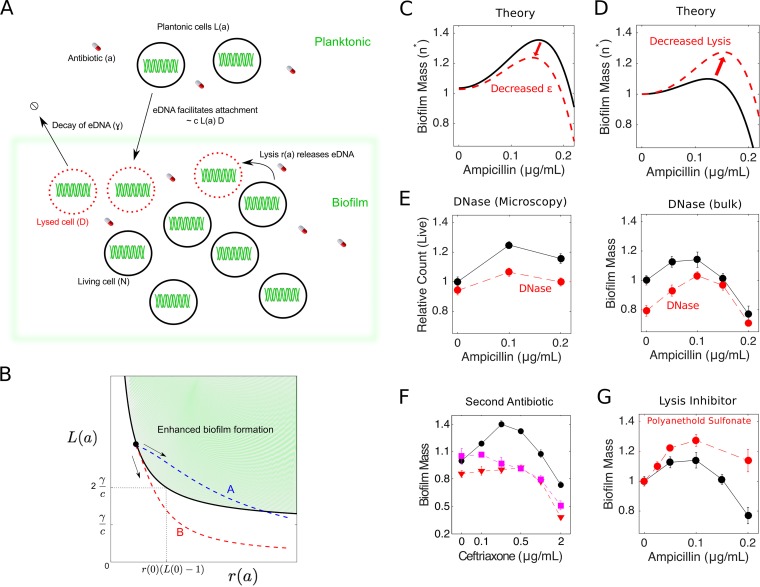
Mathematical model predicts effects of DNase, non-lysis-inducing drugs, and cell lysis inhibitors. (A) Simple mathematical model that couples cell lysis to biofilm formation, describing qualitative features of antibiotic-mediated biofilm enhancement. Lysis of living biofilm cells (*N*) depends on the drug concentration *a* according to *r*(*a*). Lysed cells and dead cell material (*D*) facilitate cell adhesion and attachment from planktonic cells [*L*(*a*)]. Adhesion/attachment is presumably enhanced due to release of eDNA, which itself detaches and decays at a rate γ, and surface attachment is proportional to *L*(*a*) and *D* with a rate constant *c*. In practice, the model contains two free “effective” parameters [ε 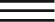 *cL*(0)/γ and *r*_01_], which can be estimated from the peak height and peak location in biofilm enhancement curves (e.g., Fig. 1 or Fig. 4). Intuitively, the parameter ε describes the effective coupling between cell lysis and biofilm induction; a peak in biofilm mass occurs at nonzero drug concentration when ε is >1. (B) Phase diagram showing the region of parameter space where enhanced biofilm formation occurs, in terms of the original model parameters. Enhanced biofilm formation is favored in regions of high lysis [*r*(*a*)] and large planktonic populations [*L*(*a*)]. Blue and red dashed lines indicate paths taken by the system as the antibiotic concentration is increased; arrows indicate the direction of increasing *a*. The blue curve (path A) exhibits enhanced biofilm formation, while the red curve (path B) does not. (C) Model predicting that perturbations that decrease ε (left) lead to decreases in the height and location of the peak in the living biofilm mass (n* 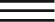 *N*/*K*) (solid line). Parameter values for ε of 1.18 ± 0.01 and *r*_01_ of 19 ± 4 in the absence of perturbation were estimated from living biofilm cell counts from confocal microscopy (Fig. 4). The dashed curve shows the predicted change in peak location and height due to perturbations that reduce the coupling ε by several percent. (D) Model predicting that perturbations that decrease cell lysis lead to increases in the height and location of the maximum living biofilm mass (n*) (solid line). Parameter values for ε of 1.09 ± 0.02 and *r*_01_ of 18 ± 6 were estimated from bulk experiments (Fig. 1). The dashed curve shows the predicted change in peak location and height due to perturbations that decrease cell lysis. (E) Relative biofilm mass (solid curves) as a function of ampicillin from confocal microscopy (left) (also see Fig. 4) and bulk experiments (right) (also see Fig. 1). Dashed curves indicate identical experiments but with DNase I added at a concentration of 0.4 mg/ml. (F) Relative biofilm mass as a function of ceftriaxone alone (circles) or ceftriaxone in combination with a constant concentration of rifampin at 0.3 μg/ml (squares) or tetracycline at 0.2 μg/ml (inverted triangles). (G) Relative biofilm mass. The solid curve is the same as in panel E, right. The dashed curves indicate identical experiments but with SPS, which inhibits cell lysis (see Fig. S3 in the supplemental material), at a concentration of 10 μg/ml. Error bars represent standard errors of the mean.

In the steady state, the living biofilm mass *N* is given by

(2) $$\frac{N}{K}\equiv n*=1+r_{0}(a)\left[L_{0}(a)-1\right]$$

where *r*_0_(*a*) = *r*(*a*)/*g* and *L*_0_(*a*) = *cL*(*a*)/γ are (rescaled) functions describing the rate of cell lysis and the number of living cells in planktonic solution, respectively, as a function of drug *a*. Equation 2 illustrates a simple balance between the biofilm-inducing and biofilm-inhibiting effects of antibiotics. To understand this trade-off, we derived a phase diagram (Fig. 5B) showing regions of enhanced biofilm formation, specifically, regions of the [*r*(*a*),*L*(*a*)] plane where n* is greater in the presence of drug than in its absence (see the supplemental material for details). Enhanced biofilm formation is favored in regions of high lysis *r*(*a*) and large planktonic populations *L*(*a*). However, lysis *r*(*a*) is expected to increase with the drug concentration, while the number of available cells in the planktonic phase *L*(*a*) is expected to decrease with the drug concentration *a* and eventually tend toward zero. The balance between these two effects determines the path taken by the system through the [*r*(*a*),*L*(*a*)] plane as drug is added. If increasing drug concentrations lead to increased lysis without a dramatic impact on planktonic cells, then the system exhibits enhanced biofilm formation (Fig. 5B, path A); if increasing drug concentrations lead to a large decrease in planktonic cells and a relatively small increase in lysis, then biofilm formation is not enhanced (Fig. 5B, path B).

In principle, the functional forms for *r*_0_(*a*) and *L*_0_(*a*) could be derived from microscopic models that describe the molecular dynamics of antibiotic-induced cell lysis and cell death. Fortunately, however, the functions *r*_0_(*a*) and *L*_0_(*a*) can also each be estimated, up to a scaling constant, by independent experimental measurements, even in the absence of a detailed molecular model. Specifically, using the data in Fig. 3A and B, we take

(3) $$\begin{array}{c} L_{0}(a)=\frac{\varepsilon }{1+a^{h}}\\ r_{0}\left(a\right)=r_{01}\left(r_{00}+a^{2}\right) \end{array}$$

where *h* = 3 describes the steepness of the dose-response curve (and is analogous to Hill coefficients used in biochemistry to describe cooperative binding between ligands), *a* is measured in units of the half-maximal inhibitory concentration of the drug (estimated as *K*_50_ = 0.38 ± 0.01 μg/ml), and *r*_00_ is 0.010 ± 0.001. It should be noted that we assume a quadratic dependence of lysis on *a* to match the experimental measurements; this should be viewed as a simple parameterization of the experimental lysis measurements and does not imply any particular mechanism. The quadratic dependence of lysis on *a* could depend on complex pharmacological and pharmacodynamic characteristics of the antibiotics, and we do not attempt to model those here.

The remaining two parameters, ε and *r*_01_, are scaling parameters that can be estimated from biofilm induction curves (e.g., Fig. 1). Because the measured value of *r*_00_ is much less than 1, we can derive approximate solutions for the location (*a*_max_) and height (*p_h_*) of the biofilm peak by using simple perturbation theory (see the supplemental material). Specifically, the peak location is given by

(4) $$a_{\max}\approx {\left[\frac{2\left(\varepsilon -1\right)}{4+\varepsilon }\right]}^{1/3}$$

and the peak height is given by

(5) $$p_{h}\approx 1+\frac{3}{5}{\left(\frac{2}{5}\right)}^{2/3}r_{0}{\left(\varepsilon -1\right)}^{5/3}$$

These expressions indicate that optimal biofilm production occurs at a nonzero concentration *a* when ε is >1, and the effect of further increasing ε is to shift the peak to higher *a* values and to increase its height. We refer to ε as an effective coupling parameter and, given the functional forms in equation 3, the value of ε alone determines whether there is a peak in biofilm formation at nonzero drug concentrations. In terms of the original model parameters, ε is given by ε = *cL*(0)/γ, where *L*(0) is the size of the planktonic cell population at zero drug concentration. Antibiotic-mediated biofilm formation occurs for ε >1 and therefore is favored with high rates of lysis-mediated adhesion (*c*), high concentrations of planktonic cells [*L*(0)], and slow degradation of eDNA (γ).

### The model predicts effects of DNase treatment, a second antibiotic, and lysis inhibitors.

While it is straightforward to estimate ε and *r*_01_ from biofilm experiments (for example, ε = 1.09 ± 0.02 and *r*_01_ = 18 ± 6, based on the bulk experiments in Fig. 1A), it is more instructive to consider the qualitative predictions of the model as parameters are varied. Our model predicts that perturbations that decrease ε would decrease the peak height (Fig. 5B; also see Fig. S2, bottom left). Perturbations that decrease cell lysis would shift *r*_0_(*a*) to *r*_0_(*a*) − β, with β being a positive constant; equivalently, decreasing lysis would shift *r*_00_ to *r*_00_ − (β/*r*_01_). While the latter effects would not be evident at the level of the approximate equations (equations 4 and 5), we can evaluate the predicted effects numerically (Fig. 5C; also see Fig. S2, bottom right) or by looking at higher-order terms in the approximation (see the supplemental material). Decreasing lysis is predicted to shift the peak location to higher drug concentrations and, somewhat surprisingly, leads to an increase in the magnitude of biofilm induction (that is, an increase in the height of the peak relative to biofilm mass in the drug-free case). In other words, a higher concentration of antibiotic is needed to achieve sufficient cell lysis to induce increased biofilm production.

To test these predictions experimentally, we first repeated both bulk and microscopy experiments in the presence of DNase I. Because eDNA has been implicated as the molecular conduit linking cell lysis to biofilm formation, we expect DNase treatment to decrease ε by effectively increasing the decay rate γ. Indeed, biofilms treated with DNase exhibited smaller peaks (Fig. 5D). The model also predicts a slight shift in the location of the peak, but the resolution of the experimental data is insufficient to evaluate that prediction quantitatively. A second way to decrease ε would be to decrease the number of living cells in the planktonic phase [*L*(0)]. One possibility is to treat the cells with a second (non-lysis-inducing) antibiotic, which is expected to decrease the steady-state density of cells in the liquid phase. Consistent with this prediction, we observed that treatment with a cell wall synthesis inhibitor (ceftriaxone) along with a second, non-lysis-inducing drug (tetracycline or rifampin) decreased the height of the peak to almost zero (Fig. 5E).

Next, to test the prediction that decreasing lysis leads to an increase in relative peak height and location, we repeated the biofilm experiment in the presence of sodium polyanethol sulfonate (SPS). SPS is a common anticoagulant used in clinical blood cultures and is known to have a positive effect on bacterial survival (31). It has been shown to inhibit cell lysis in staphylococci by suppressing the activity of autolytic wall systems (27, 32, 33). To verify that SPS inhibited cells lysis in our system, we measured lysis as a function of SPS concentration using an ATP-based luminescence assay (29); our data suggest that SPS inhibits cell lysis by approximately 40% in the absence of drug, at the concentrations used (Fig. S3). We found that treatment with the lysis inhibitor appeared to shift the peak to slightly higher drug concentrations and to increase the magnitude of biofilm enhancement, again consistent with predictions from the model.

## DISCUSSION

Our work demonstrates that biofilm formation in E. faecalis is enhanced by subinhibitory concentrations of cell wall synthesis inhibitors but not by inhibitors of protein, DNA, folic acid, or RNA synthesis. Enhanced biofilm formation is associated with increased cell lysis and increases in eDNA and eRNA. We observed similar enhancement effects when cultures were treated with nonantibiotic chemicals that induce similar amounts of cell lysis. To quantify the trade-off between drug toxicity and the beneficial effects of cell lysis, we developed a simple mathematical model that predicts changes to drug-induced biofilm formation due to external perturbations that reduce eDNA levels, reduce the number of living cells in the planktonic phase, or inhibit cell lysis. Our model suggests that antibiotic-induced biofilm formation occurs when the drug-induced increase in cell lysis is sufficiently large, relative to the drug-induced decrease in living cells in the planktonic phase.

Subinhibitory concentrations of antibiotics have been reported to promote biofilm formation in multiple species via a range of different mechanisms (14, 16). However, relatively little is known about drug-induced biofilm formation in E. faecalis. Subinhibitory antibiotic concentrations have been shown to affect the physioelectrical (34) and adhesion (35) behavior of E. faecalis. In addition, low concentrations of tigecycline have been shown to reduce biofilm formation even when the growth of planktonic cells is not significantly affected (36). To our knowledge, however, this is the first work to describe enhancement of biofilm formation by E. faecalis due to cell wall synthesis inhibitors. In contrast, our work suggests that inhibitors of protein, DNA, folic acid, and RNA synthesis do not promote biofilm formation over a wide range of subinhibitory concentrations (although we do caution that we cannot definitively rule out the possibility of biofilm promotion at higher concentrations, perhaps via different mechanisms).

Our results are consistent with the established role of eDNA in biofilm formation and may be applicable to drug-induced biofilm formation in other species, most notably S. aureus (27). However, it is possible that other mechanisms may also contribute, at least in part, to our results. For example, recent work has shown that eDNA is prevalent in biofilms even at the early developmental stages when cell lysis is minimal (29), and it is possible that subinhibitory drug concentrations increase eDNA levels through similar nonlytic mechanisms. In addition, it is well known that sub-MIC levels of antibiotic can dramatically alter gene expression profiles in bacteria (37–39), indicating that biofilm enhancement may arise from a complex combination of multiple factors. Finally, we note that live-dead cell staining results should be interpreted with some caution, because uptake of various stains may be variable (40–44). Nevertheless, our results are promising because they suggest that, at least under the experimental conditions assessed here, a simple conceptual (and mathematical) model is sufficient to describe and to predict the primary effects of drug exposure.

Despite the model's success, it is without question a dramatic oversimplification of the complex biofilm formation process. Computational models of biofilm formation are powerful tools for understanding the dynamics and evolution of complex communities (45–47), and detailed models may contain dozens or even hundreds of microscopic parameters; however, even the most elaborate mathematical models neglect biological details at some level. Our approach was not to develop a detailed microscopic model but rather to develop a simple minimal model to help intuitively explain and to predict the trade-offs between antibiotic efficacy and beneficial cell lysis at the population level. Linking our model with more detailed agent-based simulations may help us further understand the potential role of spatial structure and heterogeneity in drug-induced biofilm formation. For example, recent work has shown that, in the absence of drug, E. faecalis biofilm formation depends on phenotypic bistability in gene expression, giving rise to lysis-susceptible and lysis-inducing subpopulations (19–23). It would be interesting to further explore the interplay between this multimodal population structure and the drug-induced lysis observed in this work.

Our work also raises intriguing questions about how genetic resistance determinants might spread in biofilm populations, even in the absence of the strong selection pressure of high drug concentrations. A quantitative understanding of biofilm formation may also inspire new optimized dosing protocols, similar to those used in, for example, references 48–50, and the current model could be easily extended to investigate the effects of clinically realistic antibiotic dosing regimens. In the long run, these results may lay the groundwork for improved systematic design of biofilm-specific therapies (51, 52).

## MATERIALS AND METHODS

### Bacterial strains and media.

Experiments were performed with Enterococcus faecalis V583, a fully sequenced clinical isolate (53), and strain OG1RF, which was derived from human oral isolate OG1 (54). For each experiment, starting cultures (3 ml) were inoculated from a single colony on BHI agar plates (1.5% [wt/vol] bacteriological agar) and were grown overnight in BHI medium or TSB at 37°C without shaking.

### Drugs.

The antibiotics used in this study are listed in Table 1. Antibiotic stock solutions were sterilized by passage through 0.22-μm filters, aliquoted into daily-use volumes, and kept at −20°C or −80°C for no more than 3 to 6 months. All chemicals and media were purchased from Sigma-Aldrich or Fisher Scientific unless stated otherwise.

**TABLE 1:**
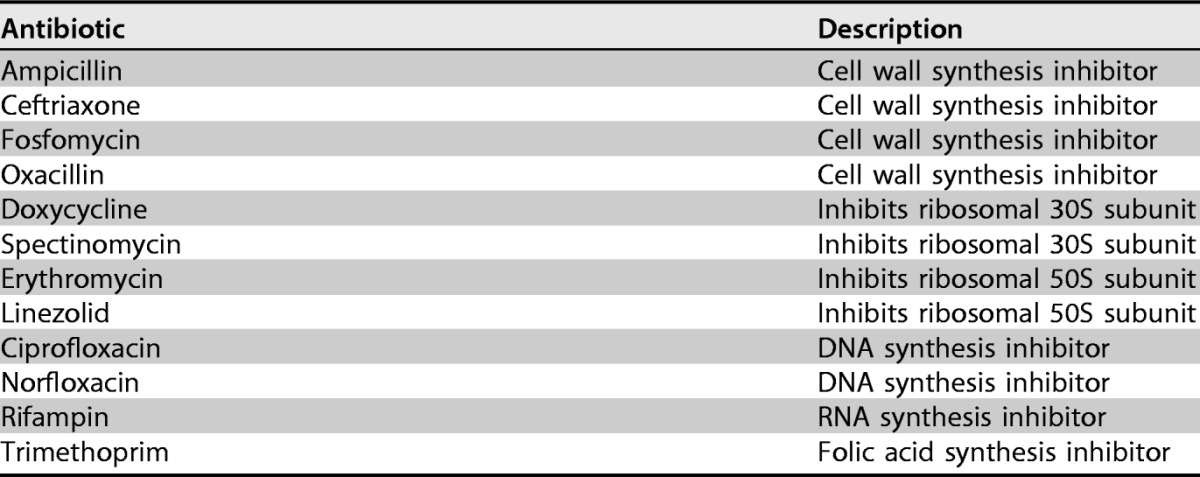
Antibiotics used in this study

### Growth curves for Enterococcus faecalis.

Overnight cultures were diluted 100-fold into fresh BHI medium, and then 200 μl of diluted culture was added to each well of a 96-well clear-bottomed plate. Different concentrations of antibiotics were then added to each well, and time series of the optical density at 600 nm (OD_600_) were measured at 15-min intervals for 24 h at 30°C, using an EnSpire multimodal plate reader, in a temperature-controlled warm room.

### Microtiter plate biofilm assay.

We measured biofilm mass in bulk assays using a well-established crystal violet staining assay (55, 56). Overnight cultures were diluted 100-fold into fresh BHI medium, and then 100 μl of diluted culture (along with appropriate concentrations of antibiotics, if relevant) was added to each well of a 96-well flat-bottomed polystyrene microtiter plate (Cellstar; Greiner Bio-One). The plate was incubated for 24 h at 37°C without shaking. After incubation, supernatant from liquid cultures was removed by gently inverting the plate, shaking it, and patting it on paper towels. Wells were then gently washed with phosphate-buffered saline (PBS). To fix the biofilm on the plate, 125 μl of 96% ethanol was added to each well and allowed to incubate for 20 min. Ethanol was then removed and the plate was dried at room temperature for 30 min. After drying, 125 μl of 0.5% crystal violet was added to stain the biofilm mass. After 30 min, plates were washed multiple times with fresh PBS. Plates were then turned upside down and dried for 1 h. Finally, 125 μl of 30% acetic acid was added to each well in order to dissolve the biofilm. Solutions were transferred to a new 96-well plate, and readings of absorbance at 590 nm were taken using an EnSpire multimodal plate reader. For each treatment, we performed 6 to 12 replicates.

### ATP detection assay.

To measure cell lysis, we used the luminescence assay described in reference 29 to measure increases in extracellular ATP levels using a commercial ATP determination kit (Molecular Probes). Prior to measuring cell lysis, biofilms were grown for 24 h with different concentrations of antibiotics, as described previously, in a 96-well plate. We then washed the plate twice with nuclease-free water and removed excess liquid. After washing, 10 μl of nuclease-free water was added to each well and biofilms were scraped by using inoculation loops or pipette tips. Solutions were transferred to a new 96-well white polystyrene plate (Nunc F96 MicroWell; Thermo Scientific), and 90 μl of ATP standard assay solution from the ATP determination kit (Molecular Probes) was added to each sample. Luminescence was measured with a plate reader.

### Confocal laser scanning microscopy.

Bacterial cultures (total volume of 200 μl, with appropriate drug treatment) were grown in replicates of 4 in 16-well chambered coverglass vessels. After incubation for 24 h, liquid was removed and the plate was washed twice with filtered Millipore water and then stained for 20 min using the LIVE/DEAD BacLight bacterial viability kit (Molecular Probes). After staining, the liquid was removed and a coverglass was affixed to the top of the chamber.

The biofilms were imaged using a Zeiss LSM700 confocal laser scanning microscope (with a 40×, 1.4-numerical-aperture objective; Zeiss), with laser lines at 488 nm and 555 nm being used for excitation. For each well, four image stacks (160 by 160 μm) spanning 20 to 30 μm (vertically), at 1-μm intervals, were taken at four separate (*x*,*y*) locations on the coverslip, giving a total of 16 biofilm images per condition. To analyze images, we split them into red and green channels, set the threshold for each slice individually using automated thresholding algorithms in ImageJ, and then used a watershed algorithm to segment cells and to determine the location and size of each cell type (live or dead) in each slice. Cell counts per slice were averaged over three well-separated slices (to avoid double counting of cells in adjacent slices) in the middle portion of each biofilm and over all 16 images per condition.

### Extracellular DNA/RNA extraction.

Biofilms were grown in triplicate with 0, 0.1, or 0.2 μg/ml ampicillin in 6-well polystyrene plates, with a total volume of 5 ml for each well. After 24 h, we discarded the liquid and washed the plate twice with PBS before adding 1 ml of 1× Tris-EDTA (TE) buffer (10 mM Tris-Cl, 1 mM EDTA [pH 8.0]) and scraping the biofilms from the bottom of the plates.

After harvesting of the biofilms, cells were removed by centrifugation and the supernatant was purified by using only the binding and washing steps of the QIAprep Spin Miniprep kit, according to the manufacturer's instruction. Five volumes of buffer PB was added to 1 volume of supernatant and mixed, and 800 μl of solution was transferred to a spin column and centrifuged at 13,000 rpm for 1 min. A volume of 0.5 ml of buffer PB was added to wash the spin column, followed by centrifugation for 1 min. Then, 0.75 ml of buffer PE was added to the spin column, and the column was centrifuged again for 1 min. The flowthrough fraction was discarded, and the residual was removed by centrifuging the spin column for an additional 1 min. The column was then transferred to a new 1.5-ml microcentrifuge tube and 30 μl of buffer EB was added to the center of the spin column. After 1 min, the column was centrifuged for 1 min to elute DNA. DNase I or RNase was added to the same treatment samples as controls.

### Agarose gel electrophoresis.

Gel trays and all related tools were rinsed with nuclease-free water, and samples were loaded on a 1% agarose gel with 1× Tris-acetate-EDTA (TAE) buffer (pH 8.4) containing an appropriate volume of SYBR Safe stain. The gel was run at 120 V for 40 min in 1× TAE buffer. DNA or RNA fragments were visualized under UV light from a UV transilluminator. To analyze images, ImageJ software was used to subtract the background and to perform intensity analysis for different lanes. Identically sized regions were selected for different lanes, and a profile plot of each lane was drawn. A straight line across the base of the peak was drawn to enclose the peak, and the wand tool was used to select each peak and to measure percentages of relative densities.

## Supplementary Material

Supplemental material
