# Supplementary material for: Interplay between Antibiotic Efficacy and Drug-Induced Lysis Underlies Enhanced Biofilm Formation at Subinhibitory Drug Concentrations
Source: Antimicrob Agents Chemother. 2017 Dec 21;62(1):e01603-17. doi: 10.1128/AAC.01603-17 (PMC5740344; doi:10.1128/AAC.01603-17)
Supplement: Supplemental material [file supp_62_1_e01603-17__index.html]

Interplay between Antibiotic Efficacy and Drug-Induced Lysis Underlies Enhanced Biofilm Formation at Subinhibitory Drug Concentrations — Supplemental material 

# Interplay between Antibiotic Efficacy and Drug-Induced Lysis Underlies Enhanced Biofilm Formation at Subinhibitory Drug Concentrations

## Supplemental material

- Supplemental file 1 -

  Text S1

  PDF, 573K
