# Supplementary material for: Interplay between Antibiotic Efficacy and Drug-Induced Lysis Underlies Enhanced Biofilm Formation at Subinhibitory Drug Concentrations
Source: Antimicrob Agents Chemother. 2017 Dec 21;62(1):e01603-17. doi: 10.1128/AAC.01603-17 (PMC5740344; doi:10.1128/AAC.01603-17)
Supplement: Supplemental material [file AAC.01603-17_zac001186780s1.pdf]

SI: Interplay between antibiotic efficacy and drug-induced lysis underlies enhanced biofilm formation at subinhibitory drug concentrations

Wen Yu,[1] Kelsey Hallinen,[2] and Kevin B. Wood[1,2]
[1)] *Department of Physics, University of Michigan, Ann Arbor, Michigan 48109, USA*
[2)] *Department of Biophysics, University of Michigan, Ann Arbor, Michigan 48109, USA*

This supporting material contains a detailed description of the mathematical model and analysis as well as three supplemental figures, including experimental measurements of growth and cell lysis in planktonic cultures and gel images of eDNA (Figure S1), analysis of approximate solutions to the mathematical model (Figure S2), and experimental measurements of cell lysis in biofilms exposed to a chemical lysis inhibitor (Figure S3). The ordering of the figures follows the order in which they are referenced in the main text.

I. MATHEMATICAL MODEL

To model lysis-induced biofilm formation, we consider a simple model given by

$$\begin{aligned} \frac{\partial N}{\partial t} &= g\left(1-\frac{N}{K}\right)N - rN + cLf(D) \\ \frac{\partial D}{\partial t} &= rN - \gamma D \end{aligned} \tag{1}$$

where N is the living cell mass in the biofilm, D is the mass of lysed (dead) cells and dead cell material, and L is the number of living cells in the planktonic media. In the first equation, the first term describes logistic growth (with per capita growth g and carrying capacity $K > 0$), the second describes cell death (lysis) with rate $r \geq 0$, and the last term describes the increase in biofilm mass due to surface attachment of living cells in the planktonic phase. When $f(D)$ is a constant, cells attach to the biofilm at a rate proportional to the number of cells in the planktonic phase ($L > 0$) times a rate parameter $c > 0$; more general choices for $f(D)$ couple biofilm induction to cell lysis, which we show below is required to achieve a peak in N as a function of lysis. In the second equation, the first term accounts for cell lysis and the second term describes a decay of dead (lysed) cell material due to, for example, detachment from the biofilm. The model includes two parameters, r and L, that depend on drug concentration, which we call a. In what follows, we begin our analysis under mild assumptions on $r(a)$ and $L(a)$. Then, for a more detailed analysis, we resort to specific functional forms which can be estimated, up to a scaling constant, directly from experimental data.

A. Biofilm formation uncoupled from lysis

We first consider a simple case where biofilm formation is uncoupled from cell lysis, i.e. $f(D) =$ constant (which we subsume into the constant c without loss of generality). In this case, Equation 1 can be written in terms of dimensionless variables $n = N/K$, $d = Dg/(Kr)$, and rescaled time $\tau = tg$ as

$$\begin{aligned} \frac{\partial n}{\partial \tau} &= (1-n)\,n - r_0 n + L_0 \\ \frac{\partial d}{\partial \tau} &= n - \gamma_0 d \end{aligned} \tag{2}$$

where $r_0 = r/g$, $L_0 = cL/(gK)$, and $\gamma_0 = \gamma/g$. In the steady state, we have

$$\begin{aligned} n^* &= \frac{1}{2}\left(1 - r_0 + \sqrt{(1-r_0)^2 + 4L_0}\right) \\ d^* &= \frac{1}{2\gamma_0}\left(1 - r_0 + \sqrt{(1-r_0)^2 + 4L_0}\right) \end{aligned} \tag{3}$$

where we have kept only the physically meaningful (positive) root. It is straightforward to show that this steady state is always a stable fixed point (tr$J < 0$ and det$J > 0$, where J is the Jacobian of the system in Equation 2 evaluated at (n^*, d^*)).

It is intuitively clear that this model does not exhibit a non-zero peak in n^* as a function of antibiotic a. Recall that the dependence on a arises from $r_0(a)$ and $L_0(a)$, which are functions of drug concentration. If we make the physically reasonable assumptions that, for $a > 0$, $r_0'(a) > 0$ (lysis increases with drug) and $L_0'(a) < 0$ (planktonic cells decrease with drug) –both of which are consistent with experimental measurements–the derivative of $n^*(a)$ is always negative. Specifically, we have

$$\frac{\partial n^*(a)}{\partial a} = \frac{1}{2}\left(r_0'(a)(\lambda - 1) + \frac{2L_0'(a)}{\sqrt{4L_0(a) + (r_0(a)-1)^2}}\right) \tag{4}$$

where primes indicate differentiation with respect to a and $\lambda = \frac{r_0(a)-1}{\sqrt{4L_0(a)+(r_0(a)-1)^2}}$. Because $|\lambda| \leq 1$, both terms are negative, indicating that $n^*(a)$ is always decreasing and cannot exhibit a maximum for $a > 0$.

B. Biofilm formation coupled to lysis

To capture experimental observations in a minimal model, we consider Equation 1 with $f(D) = D$, so that

the mass of dead (lysed) cells is coupled to living biofilm mass. We can write Equation 1 in terms of rescaled variables $n = N/K$, $d = Dg/(Kr)$, and $\tau = tg$ as

$$\begin{aligned} \frac{\partial n}{\partial \tau} &= (1-n)\,n - r_0 n + \gamma_0 L_0 r_0 d \\ \frac{\partial d}{\partial \tau} &= n - \gamma_0 d \end{aligned} \tag{5}$$

where $r_0 = r/g$, $L_0 = cL/\gamma$, and $\gamma_0 = \gamma/g$. In the steady state, we have

$$\begin{aligned} n^* &= 1 + r_0\,(L_0 - 1) \\ d^* &= \frac{1}{\gamma_0}\,(1 + r_0\,(L_0 - 1)) \end{aligned} \tag{6}$$

We restrict our analysis to the physically-meaningful regime $r_0(1 - L_0) < 1$, where the steady state values n^* and d^* are positive definite. In this regime, the steady state solution Equation 6 is always a stable fixed point ($\mathrm{tr}J < 0$ and $\det J > 0$, where J is the Jacobian of the system in Equation 5 evaluated at (n^*, d^*)).

To look for enhancement of biofilm (living) mass as a function of a, we derive a simple phase diagram illustrating the region of parameter space where $n^*(a) > n^*(0)$; that is, we look for regions where living biofilm mass is higher in the presence of drug than in its absence. To do so, we plot the curve given by

$$n^*(a) - n^*(0) = 0, \tag{7}$$

which after rearrangement becomes

$$L_0(a) = \frac{r_0(0)(L_0(0) - 1)}{r_0(a)} + 1. \tag{8}$$

In terms of the original model parameters, we have

$$L(a) = \frac{\gamma r(0)(L(0) - 1)}{c}\frac{1}{r(a)} + \frac{\gamma}{c}. \tag{9}$$

Equation 9 shows that $L(a) = \frac{2\gamma}{c}$ for $r(a) = r(0)(L(0) - 1)$ and asymptotically approaches $\frac{\gamma}{c}$ as $r(a) \to \infty$. The curve separates regions where drug decreases biofilm formation from those regions with enhanced biofilm formation (Figure 5). Enhanced biofilm formation therefore depends on the path taken through $(r(a), L(a))$ space as a is increased from 0. In words, enhanced biofilm formation is favored by high lysis $r(a)$ and large planktonic populations $L(a)$, and the specific dependence of these functions on drug concentration determines whether drug will increase or decrease biofilm mass.

To find the location of the biofilm peak, we differentiate Equation 6 with respect to a, leading to

$$\frac{\partial n^*(a)}{\partial a} = r_0(a)L_0'(a) + (L_0(a) - 1)\,r_0'(a) = 0. \tag{10}$$

The existence and location of an optimum is determined by properly scaled functions–and the corresponding first derivatives–describing lysis ($r_0(a)$) and the decay of living cells in the planktonic phase ($L_0(a)$) as a function of drug. Both of these functions can be independently measured–up to a scaling constant– in our experiments. In turn, these two scaling constants become free parameters which can be estimated, for example, from the peak height and peak location in our biofilm experiments.

To make further analytical progress, we assume that

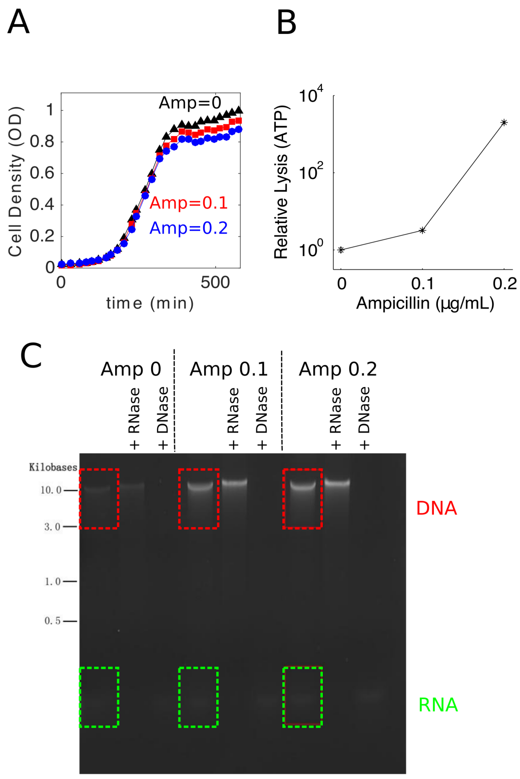

FIG. S1. A. Growth curves of planktonic populations (V583, BHI) for ampicillin at 0 (black triangles), 0.1 μg/mL (red squares), and 0.2 μg/mL (blue circles). OD is normalized so that OD=1 at the last time point in the absence of drug. B. Subinhibitory concentrations of ampicillin increase cell lysis in planktonic populations. Error bars are $\pm$ standard error of the mean over replicates. Lysis is measured by ATP-based luminescence assay (Methods). C. Gel image following electrophoresis of nucleic acid isolated from biofilms. eDNA and eRNA are quantified within the red and green dashed boxed regions, respectively. For example, eDNA was considered to be bands larger than 3.0 kilobases. Additional lanes show effects of treatment with RNase or DNase; these treatments were used to determine the approximate regions corresponding to eDNA and eRNA, respectively. This experiment was performed three times on three different days; while the quantitative results (e.g. total eDNA/eRNA intensity) vary from day-to-day, the trends are always similar to those shown in Figure 3C. Note that eRNA bands are considerably lighter than eDNA bands.

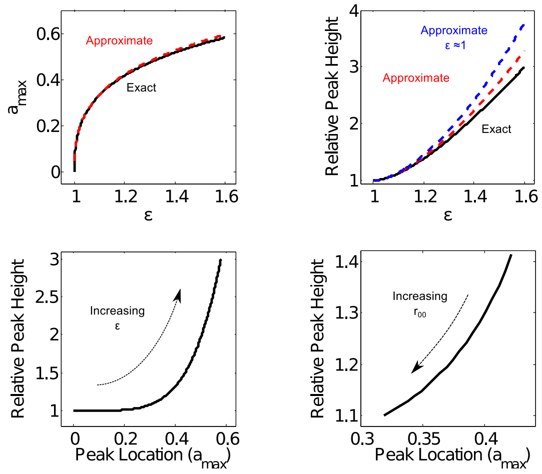

FIG. S2. Changes in ϵ and r_{00} shift peak location and peak height. Top left: Approximate equation for peak location (Equation 18, red dashed) and exact value (black). Top right: Approximate equation for peak height (Equation 19, red dashed), $\epsilon \approx 1$ expansion (Equation , blue dashed) and exact value (black). Bottom left: Peak height vs. peak location (exact) for $1 \leq \epsilon \leq 1.6$. Bottom right: Peak height vs. peak location (exact) for $0 \leq r_{00} \leq 0.2$. Parameters r_{00}, r_{01} and ϵ were chosen to match the range observed in experiments. $r_{01} = 20$ for all panels. $r_{00} = 0.01$ for top panels and bottom left panel. $\epsilon = 1.2$ for bottom right panel.

$r(a)$ and $L(a)$ take the following functional forms

$$\begin{aligned} r_0(a) &= r_{01}(r_{00} + a^2) \\ L_0(a) &= \frac{\epsilon}{(1 + a^h)} \end{aligned} \tag{11}$$

where r_{00} and r_{01} describe the increase in lysis as a function of a, ϵ is a positive definite parameter that captures the effective coupling between biofilm formation and cell lysis, h is a hill coefficient, and a is measured in units of the drug's half-maximal inhibitory concentration (IC50). Based on experimental measurements (Figure 3), we estimate $r_{00} = 0.010 \pm 0.001 << 1$, $h = 3.2 \pm 0.2$, and the drug's IC50 is given by 0.38 ± 0.01 μg/mL. For mathematical simplicity, we take $h = 3$ in what follows. As we will see, the remaining two parameters (ϵ and r_{01}) determine the location and the height of the peak in biofilm production as a function of a.

Plugging Equations 11 into Equation 10 yields a nonlinear equation that can be solved numerically to yield the peak location a_{max}. Specifically, we have $r_0'(a) = 2r_{01}a$ and $L_0'(a) = -\frac{\epsilon 3a^2}{(1+a^3)^2}$, which leads to

$$\begin{aligned} -r_{01}(r_{00} + a_{max}^2)\left(\frac{\epsilon 3a_{max}^2}{(1 + a_{max}^3)^2}\right) + \\ 2a_{max}r_{01}\left(\frac{\epsilon}{1 + a_{max}^3} - 1\right) = 0. \end{aligned} \tag{12}$$

We simplify the above equation by multiplying both sides by $-(1 + a_{max}^3)^2/r_{01}$, leading to

$$2a_{max}^6 + (4 + \epsilon)a_{max}^3 + 3\epsilon r_{00}a_{max} + 2(1 - \epsilon) = 0, \tag{13}$$

where we have factored out the $a_{max} = 0$ solution. Equation 13 has $a_{max} > 0$ solutions only when $\epsilon > 1$. Because we expect this peak to occur in the subinhibitory regime of antibiotic concentration, we assume $a << 1$ and ignore the sixth order term to give

$$a_{max}^3 + \delta a_{max} + \omega = 0 \tag{14}$$

with $\delta \equiv \frac{3\epsilon r_{00}}{4+\epsilon}$ and $\omega \equiv \frac{2(1-\epsilon)}{(4+\epsilon)}$. Since r_{00} is estimated to be on the order of 10^{-2}, we assume $\delta << 1$ and expand a_{max} in a power series as

$$a_{max} = a_0 + a_1\delta + ... \tag{15}$$

Subbing this expression into Equation 14 and equating like powers of δ, we have

$$a_0 = (-\omega)^{1/3} = \left(\frac{2(\epsilon - 1)}{4 + \epsilon}\right)^{1/3} \tag{16}$$

and

$$a_1 = -\frac{1}{3a_0} = -\frac{1}{3\left(\frac{2(\epsilon-1)}{4+\epsilon}\right)^{1/3}}. \tag{17}$$

To first order in δ, then, the peak location is given by

$$a_{max} = \left(\frac{2(\epsilon - 1)}{4 + \epsilon}\right)^{1/3} - \frac{\epsilon r_{00}}{(4 + \epsilon)^{2/3}\left(2(\epsilon - 1)\right)^{1/3}} \tag{18}$$

In this limit, the peak occurs for nonzero a_{max} when $\epsilon > 1$, and increasing ϵ further shifts the peak to higher antibiotic concentrations. Interestingly, Equation 18 also shows that increasing the native level of cell lysis (i.e. increasing r_{00}) is expected to shift the peak to lower values of a.

We can also plug Equation 18 into the expression for n^* (Equation 6) to get an expression for the peak height, p_h. The full expression is cumbersome, even to first order in δ, but the 0th order approximation ($\delta = 0$) is given by

$$p_h = 1 + \frac{2^{2/3}(\epsilon^2 + \epsilon - 2)r_{01}}{2 + 3\epsilon}\left(\frac{\epsilon - 1}{4 + \epsilon}\right)^{2/3} \tag{19}$$

For ϵ just above 1, the expression can be expanded to yield

$$p_h \approx 1 + \frac{3}{5}\left(\frac{2}{5}\right)^{2/3} r_{01}(\epsilon - 1)^{5/3} \tag{20}$$

which makes it clear that increasing ϵ increases the peak height.

Figure S2 shows that the approximate solutions derived above capture the ϵ dependence of relative peak height and peak location well (top panels). The model predicts that increasing ϵ leads to an increase in both peak height and peak location (bottom left panel). On the other hand, increasing r_{00} leads to a decrease in both relative peak height and peak location (bottom right

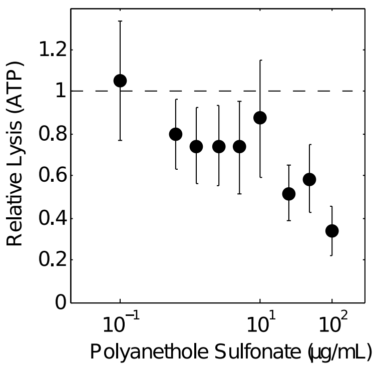

FIG. S3. Sodium Polyanethole Sulfonate (SPS) reduces cell lysis in biofilms. The figure shows cell lysis (relative to untreated cells) as a function of SDS concentration as measured by ATP luminescence assay (see Methods). Error bars are ± standard error of the mean from eight replicates.

panel). It's instructive to consider these trends in terms of the original model parameters. Rewriting the second equation in Equation 11 in terms of the original model parameters, we have

$$\frac{cL(a)}{\gamma} = \frac{\epsilon}{(1+a^h)}. \qquad (21)$$

Hence $\epsilon = \frac{cL(0)}{\gamma}$. Increasing ϵ therefore corresponds to 1) increasing the coupling between biomass material and lysis (c), 2) decreasing the decay rate of lysed cell material (γ), and/or 3) increasing the number of living cells in solution ($L(0)$). In terms of experimental perturbations, ϵ could be decreased by treating biofilms with DNase, which underlies the hypothesized biological coupling between lysis and biofilm formation. This treatment would therefore be expected to increase γ, the decay rate of lysed cell material (i.e. eDNA). A second way of decreasing ϵ would be to decrease the number of living cells in planktonic phase ($L(0)$). One possibility is to treat the cells with a second (non-lysis-inducing) antibiotic; indeed, treatments with tetracycline and rifampicin decrease the height of the peak to almost zero (Figure 5). Decreasing r_{00} corresponds to decreasing the basal level of cell lysis (for example, by adding a cell lysis inhibitor, Figure 5).
